# Controlling the Timing of Gene Expression during Organ Development

**DOI:** 10.1371/journal.pbio.0020409

**Published:** 2004-10-19

For more than 2,000 years, from the time of Aristotle onwards, it was thought that the complete body plan of human beings (and that of other animals) was present in the fertilized egg. During pregnancy, a preformed miniature human being, or homunculus, grew bigger and bigger; development was simply a process of growth.

Then, in the mid-18th century, Carl Friedrich Wolff described how the chick gut, basically a tube, forms from an initially flat sheet of cells, overthrowing at a stroke the preformation theory of embryology. We now know that development is a complex series of coordinated processes that transforms the amorphous ball of cells produced from the fertilized egg by cell division into an intricate body containing numerous specialized tissues and organs. And we are beginning to understand how a wide array of transcription factors—proteins that bind to regulatory sequences within genes to control their expression—guide the sequential stages involved in development. It seems that these factors form regulatory networks that control the temporal and spatial waves of gene expression that underlie and are required for organized body building.

Susan Mango and her colleagues are studying the role of transcription factors in controlling organ development. The organ they are studying—the pharynx of the nematode worm—is relatively simple. This muscular tube, which passes bacteria (the food of this small soil-dwelling organism) from the mouth to the midgut, contains fewer than 100 cells of only seven different types.

To get an overall picture of the regulatory sequences within genes that are involved in the temporal control of pharyngeal development, the researchers identified 339 candidate pharyngeal genes by comparing gene expression profiles in mutant worm embryos that had excess pharyngeal cells with those in mutant embryos lacking pharyngeal cells. Then, by referring to a database that details gene expression patterns in nematode worms and embryos, the researchers classified 37 of their candidate genes as having early-onset expression and 34 as having late-onset expression.

Next, the scientists carefully examined the DNA sequence of each gene for candidate regulatory regions that might contribute to its temporal regulation. Of nine candidate motifs revealed by this search, six functioned as regulatory sites in in vivo assays. The researchers estimated that these six elements, together with sites that bind PHA-4—a member of a family of transcription factors that are important in digestive tract development in many animals—account for the timing of onset of expression of about half of the nematode's pharyngeal genes. Finally, the researchers used combinations of the newly discovered temporal regulation sites and PHA-4 sites in a genome-wide search that predicted pharyngeal genes and their time of onset of expression with greater than 85% accuracy.

**Figure:**
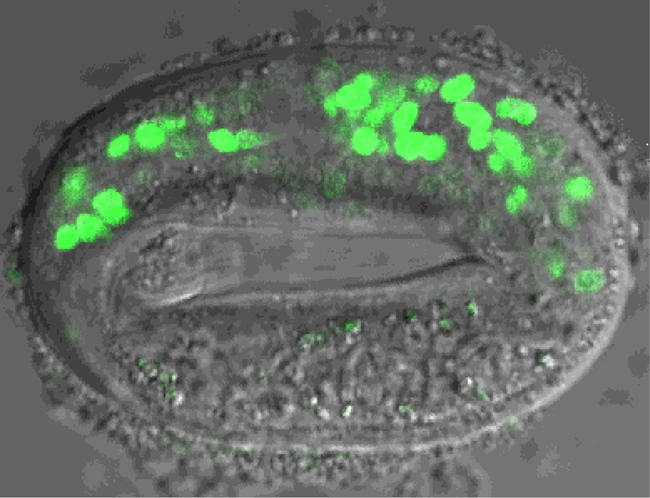
Fluorescent reporter genes expressed in the developing C. elegans foregut

From these results and those of previous studies, Mango and her colleagues propose a model to explain how the temporal control of pharyngeal gene expression needed for pharynx development is achieved. The earliest time for pharyngeal gene expression, they suggest, is determined by how well PHA-4 sticks to a particular gene's binding site. However, gene expression only occurs if other factors that bind to the regulatory sites are also present, and the exact combination of these factors determines which gene is active at any given time. The identity of these factors remains to be discovered. Nevertheless, at least for this simple organ, we now have a much better idea of how the complex process of organ formation is controlled at a molecular level, and it is likely that similar regulatory networks will underlie the formation of other organs as well.

